# Acute *Haemophilus parainfluenzae* endocarditis: a case report

**DOI:** 10.4076/1752-1947-3-7494

**Published:** 2009-07-16

**Authors:** Leonidas Christou, Georgios Economou, Anastasia K Zikou, Kaiti Saplaoura, Maria I Argyropoulou, Epameinondas V Tsianos

**Affiliations:** 1 1st Department of Internal Medicine and Hepato-Gastroenterology, Medical School, University of Ioannina, Ioannina, 45110, Greece; 2 Department of Radiology, Medical School, University of Ioannina, Ioannina, 45110, Greece

## Abstract

**Introduction:**

Numerous pathogens can cause infective endocarditis, including *Haemophilus parainfluenzae*. *H. parainfluenzae* is part of the *H. aphrophilus, Actinobacillus actinomycetemcomitans*,* Cardiobacterium hominis*,* Eikenella corrodens*, and *Kingella kingae* group that may cause about 3% of the total endocarditis cases, and is characterized by a subacute course and large vegetations.

**Case presentation:**

Acute *H. parainfluenzae* endocarditis developed in a 54-year-old woman, with no underlying predisposing factors. The patient presented with fever of 3 days duration and a severe headache. Magnetic resonance imaging of the brain revealed multiple cerebral emboli with hemorrhagic foci. Upon suspicion of endocarditis, cardiac transesophageal ultrasonography was performed and revealed massive vegetations. The patient underwent emergency mitral valve replacement, and was further treated with ceftriaxone. Blood cultures grew *H. parainfluenzae* only after valve replacement, and a 6-week course of ceftriaxone was prescribed.

**Conclusion:**

We underline the typical presentation of large vegetations in *H. parainfluenzae* endocarditis, which are associated with embolic phenomena and resulting severity. Although the majority of the few cases reported in the literature are subacute in progress, our case further underlines the possibility that *H. parainfluenzae* endocarditis may develop rapidly. Thus, awareness of the imaging characteristics of the pathogen may enhance early appropriate diagnosis and therapeutic response.

## Introduction

Although endocarditis is a common severe medical entity for which guidelines are continuously updated [1], its etiology can sometimes be vague, implicating rare pathogens. Some of them have been categorized jointly as part of the *Haemophilus aphrophilus, Actinobacillus actinomycetemcomitans*, *Cardiobacterium hominis*, *Eikenella corrodens*, and *Kingella kingae* (HACEK) group of rare bacteria that are responsible for a small, but recognizable percentage (roughly 3%) of endocarditis cases. They are all Gram-negative bacteria belonging to the oropharyngeal microflora, and are slow-growing; their growth is enhanced by the presence of carbon dioxide [2]. Various *Haemophilus* species have been implicated in the etiopathogenesis of endocarditis, including *H. influenzae*, *H. aphrophilus*, *H. paraphrophilus*, and *H. parainfluenzae*. Of these, *H. aphrophilus* is the most common pathogen in endocarditis [3], followed by *H. parainfluenzae*[4]. We describe a patient with *H. parainfluenzae* endocarditis that followed an acute course and highlight some of the most important characteristics of this rare, but significant, entity.

## Case presentation

A 54-year-old, Greek woman was admitted to our Internal Medicine Department with a high-spiking fever accompanied by rigor. She also complained of myalgias and sore throat. The symptoms started 3 days earlier. A few hours before admission, a severe headache started and led her to seek medical advice.

Her medical history was not significant. She did not report any visits to the dentist or any dental-related disease in the recent past. She had not undergone any invasive endoscopic procedure, nor had she exhibited any symptomatology in the weeks before her admission. She was not immunocompromised and did not regularly take any medications. On clinical examination, her temperature was 39.5^0^C, and her blood pressure normal. There were no clinical indications of active pulmonary or cardiac involvement, and a brief neurological examination detected no abnormalities. Nuchal rigidity was absent. A laboratory examination revealed anemia (hemoglobin 11 g/dL), and increased erythrocyte sedimentation rate (72 mm/hour) and C-reactive protein (321 mg/L). During paraclinical work-up, the patient developed multiple purpural lesions on her legs. There was no nail hemorrhage or petechial lesions on her soles or palms on clinical examination.

**Figure 1:**
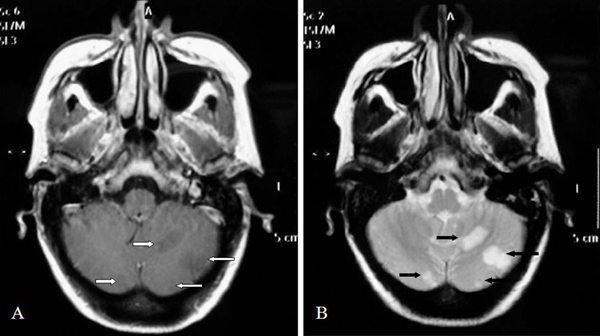
**Axial T1**(A)** and T2**(B)**-weighted images through the cerebellar hemispheres demonstrate multiple infarctions (white and black arrows)**.

Multiple cultures were drawn and a brain computed tomography (CT) scan was performed. Its findings were further assessed by magnetic resonance imaging (MRI), which revealed (Figure 1) multiple cerebellar, white matter, and sub-grey matter, low-signal T1-weighted and high-signal T2-weighted lesions. Some of these lesions exhibited no-signal T2-weighted areas, consistent with a hemorrhage.

Immediately following the MRI results, and upon strong suspicion of endocarditis leading to cerebral embolization, transesophageal echocardiography was performed, revealing two 20 mm mitral valve vegetations.

The patient was started on ceftriaxone, 2 g intravenously b.i.d. and vancomycin, 500 mg intravenously q.i.d., and mitral valve replacement surgery was performed later that day, embolization being an indication for valve replacement in infective endocarditis in the case of large vegetations such as the ones present in this patient [5]. Following valve replacement, antibiotic therapy was continued.

Four days after the valve replacement, blood cultures were positive for *H. parainfluenzae*. Throat swab specimens were cultured after the initiation of antimicrobial therapy and were negative as expected. The strain was susceptible to a variety of antibacterials, including amoxicillin-clavulanate. A decision was made to continue on ceftriaxone though, while vancomycin was discontinued. Ceftriaxone was administered, uneventfully, for six more weeks, together with gradual initiation of warfarin therapy.

## Discussion

A recent review found that less than 70 cases of *H. parainfluenzae* endocarditis have been reported in the literature, and the typical clinical pattern is that of a subacute endocarditis, developing after dental procedures in patients with pre-existing valvular disease, and carrying significant mortality (10% to 35%) [4]. However, an older review of new and historical cases found that, surprisingly for a bacterium of low pathogenicity in general, the majority of cases reported also had no predisposing factors for endocarditis development [6]. As in our patient, the mitral valve is a common site of infection, and vegetations tend to be large, again as in our patient. Early reports suggested an adverse correlation between vegetation size and outcome [7]. The size of the vegetations is further responsible for the tendency for occlusive disease associated with *H. parainfluenzae* endocarditis [8].

In our patient, the evolution of endocarditis was acute: she was retrospectively questioned for any potential symptoms that would prove a mild, subacute disease form (one that would also be consistent with the vegetations' size), but she did not recall any symptoms apart from ill-defined malaise. The patient was theoretically free of previous heart disease, and had not undergone any invasive, dental or other procedure. Thus, the probable scenario would be that of an acute endocarditis developing rapidly over the course of an acute pharyngeal infection. The patient was later evaluated for the presence of any immune defects that would partially explain her disease, but no such defect was found.

Another scenario would be that *H. parainfluenzae* was an innocent bystander, a pure pharyngeal infection that coincided with endocarditis due to another pathogen. This hypothesis could be justified on the basis that 10% of reported *H. parainfluenzae* endocarditis cases were polymicrobial in origin [4,6]. Yet, the presence of bacteremia and the absence of any other isolated pathogens rule against such a scenario.

## Conclusions

In summary, *H. parainfluenzae* is a rare, but significant cause of endocarditis, that may even be culture-negative if not actively sought after. The pathogen is a 'slow-grower, as in our patient, and may need specialized media and a high index of clinical suspicion. *H. parainfluenzae* endocarditis often causes peripheral occlusive disease due to its tendency for large vegetations; however, these may also be encountered in acute endocarditis.

## Abbreviations

CT: computed tomography; HACEK: *Haemophilus aphrophilus, Actinobacillus actinomycetemcomitans*, *Cardiobacterium hominis*, *Eikenella corrodens*, and *Kingella kingae*; MRI: magnetic resonance imaging.

## Consent

Written informed consent was obtained from the patient for publication of this case report and any accompanying images. A copy of the written consent is available for review by the Editor-in-Chief of this journal.

## Competing interests

The authors declare that they have no competing interests.

## Authors' contributions

LC, GE, KS, and EVT were the physicians in charge of the patient throughout hospitalization and follow-up. AKZ and MIA were responsible for performing, diagnosing, and discussing the imaging studies of the patient. LC prepared the manuscript draft, which was critically revised by MIA and EVT, and approved by all authors.
